# Antibodies against SARS-CoV-2 in unvaccinated children hospitalized with COVID-19: An observational cohort study of pre-Omicron and Omicron variants era

**DOI:** 10.1371/journal.pone.0297991

**Published:** 2024-02-23

**Authors:** Rattapon Uppala, Phanthila Sitthikarnkha, Kiatichai Faksri, Pope Kosalaraksa, Leelawadee Techasatian, Nattakarn Tantawarak, Sysavanh Nanthavongsa

**Affiliations:** 1 Faculty of Medicine, Department of Pediatrics, Khon Kaen University, Khon Kaen, Thailand; 2 Faculty of Medicine, Research and Diagnostic Center for Emerging Infectious Diseases (RCEID), Khon Kaen University, Khon Kaen, Thailand; 3 Faculty of Medicine, Department of Microbiology, Khon Kaen University, Khon Kaen, Thailand; Mahidol University, Faculty of Tropical Medicine, THAILAND

## Abstract

**Purpose:**

This study aimed to investigate the antibodies against SARS-CoV-2 in children hospitalized due to COVID-19 during the era of pre-Omicron and Omicron variants.

**Methods:**

This was a retrospective observational study conducted at a tertiary academic medical center in Thailand between June 2021 and August 2022. We collected the data of children aged under 18-year who were hospitalized from SARS‐CoV‐2 infection. After hospital discharge, we scheduled clinical follow-up 60 to 90 days post-infection clinical follow-up. We measured antibodies against SARS-CoV-2 anti-spike protein receptor-binding domain in the serum during a follow-up visit and compared the mean difference of antibody levels between children infected with COVID-19 during the pre-Omicron and Omicron eras.

**Results:**

A total of 119 children enrolled into the study. There were 58 and 61 children hospitalized due to COVID-19 during pre-Omicron and Omicron era, respectively. The median (interquartile range, IQR) of SARS-CoV-2 antibodies in all cases was 206.1 (87.9–424.1) U/mL at follow-up. Children infected during pre-Omicron had SARS-CoV-2 antibody levels at follow-up higher than children infected during Omicron era [mean difference 292.57 U/mL, 95% CI 53.85–531.28, *p* = 0.017). There was no difference in SARS-CoV-2 antibody levels between the children based on gender, age, co-morbidities, chest radiograph classification, or diagnosis.

**Conclusions:**

The antibodies response to SARS-CoV-2 infection was weaker during the Omicron era than previous variant of concern. Immunization strategies and policies should be implemented in children even if they had been previously infected.

## Introduction

Coronavirus Disease 2019 (COVID‐19) is caused by Severe Acute Respiratory Syndrome Coronavirus-2 (SARS‐CoV‐2) virus. It causes clinical symptoms ranging from asymptomatic to severe respiratory failure even in the era of new variants [1]. The COVID-19 pandemic continues to spread globally with emergence of new variants which have the ability to spread more rapidly than the previous variants [2]. When compared to the Delta variant, the Omicron variant BA.1/BA.2 is associated with reduced clinical severity and hospitalization risk in both children and adults [3]. Adults have significant Omicron-specific humoral responses, but data on children is limited. We previously reported that half of children infected with the Wuhan strain seroconverted, compared to adults [4].

The mortality rate of COVID-19 infection in children is less than in adults [5]. After the emergence of the Omicron variant, increased rate of SARS-CoV-2 infection was reported in children [6]. Children have lower hospitalization rates for COVID-19 than adults but serious illness and death have occurred [7]. Children hospitalized with COVID-19 were admitted to ICU in 28% to 40% of cases, invasive mechanical ventilation was necessary in 6% to 18% of cases, and up to 3% died [8,9]. SARS-CoV-2 infection in children can lead to significant consequences such as acute respiratory distress syndrome, myocarditis, and multisystem inflammatory syndrome in children (MIS-C) [8]. MIS-C was diagnosed based on the presence of antibody in individuals suspected of having obtained the virus in the community or through household contact, or on the basis of a prior history of COVID-19 infection [10].

After the initial infection, the development of antibodies is a critical factor in comprehending the dynamics of COVID-19 infection [11]. Antibodies are essential components of the immune system that can neutralize pathogens and prevent their entry into healthy cells. However, existing studies have shown conflicting results regarding the levels of antibodies and durability in non-vaccinated who experience a SAR-CoV-2 infection [12–15]. Understanding how COVID-19 antibodies work in non-vaccinated children with previous COVID-19 infections is necessary to inform public health policies and guidelines. Developing a clear understanding of how long children’s immune systems remain immune to the virus after the initial infection is important when considering the pandemic’s trajectory in the months and years ahead. Therefore, a more comprehensive assessment of SARS-CoV-2 antibody dynamics in non-vaccinated children with previous COVID-19 infections is essential. Such information may help to provide a better understanding of how COVID-19 antibodies work and how the immune system responds to the virus in children. Furthermore, it can facilitate the development of more effective public health policies and interventions to protect children and prevent the spread of COVID-19.

Due to lack of data on SARS-CoV-2 antibodies in children following Delta or Omicron infection, particularly in Asian populations, this study was conducted to determine antibodies against SARS-CoV-2 in children hospitalized due to COVID-19 during the era of pre-Omicron and Omicron variants.

## Materials and methods

### Setting

We performed a retrospective observational study between June 2021 and August 2022 at Srinagarind Hospital, Khon Kaen University, a tertiary academic medical center in Thailand’s northeastern region. This study was conducted following the Strengthening the Reporting of Observational studies in Epidemiology (STROBE) guideline. Children under the age of 18 years who had not been immunized against COVID-19 and were hospitalized with SARS-CoV-2 infection verified by real-time polymerase chain reaction (rtPCR) during the study period were enrolled in our study. After discharge from the hospital, the patients were scheduled for 60 to 90 days clinical follow-up. During the follow-up visit, the children were examined for clinical symptoms, chest radiograph, spirometry, and antibodies against SAR-CoV-2 antigen. Serum samples were collected to determine immunologic response after SAR-CoV-2 infection using antibodies against SARS-CoV-2 spike protein.

### Participants

We divided the participants into 2 cohorts, pre-Omicron, and Omicron era. The children in pre-Omicron cohort were those infected between June 2021 and December 2021, from Thailand epidemic report with the dominant variants of Delta 21A, 21J. The Omicron cohort consisted of children who were diagnose after that with the dominant variants of Omicron 21L, 22B. We excluded children who got COVID-19 vaccine before the antibody test and children who did not agree to the 60–90 days follow-up.

We used Elecsys® Anti-SARS-CoV-2 S (Roche Diagnostics International Ltd, Rotkreuz, Switzerland) to determine antibodies against SARS-CoV-2 spike protein in the serum. It is an electrochemiluminescence immunoassay (ECLIA), which has been developed for the in vitro quantitative detection of antibodies including IgG against S1- receptor binding domain antigen of SARS-CoV-2 [16]. The Elecsys® uses a recombinant protein representing the receptor binding domain (RBD) of the spike antigen in a double-antigen sandwich assay format, which favors the quantitative determination of high affinity antibodies against SARS‐CoV‐2 and The Elecsys® target the RBD of the index strain. The assay was performed following the manufacturer’s instructions. The results of SARS-CoV-2 antibody levels was reported in International Units/mL (IU/mL). A previous study indicated that the Elecsys® Anti-SARS-CoV-2 antibody levels can predict the neutralization antibodies in COVID-19 [17].

### Data collection

Clinical data of the study participants were accessed from electronic medical records between 1^st^ November and 31^st^ December 2022. We obtained demographic data including age, gender, and underlying clinical conditions. The clinical history and physical examination, detail of rtPCR, laboratory investigations including complete blood count, electrolyte, C-reactive protein, and chest radiograph results during admission due to COVID-19 were extracted from the electronic medical records. Pneumonia was defined as patients with clinical signs and symptoms of lower respiratory tract infection such as dyspnea, tachypnea, and desaturation (SpO_2_ < 94%) with abnormal chest radiograph or asymptomatic patients with abnormal chest radiograph. The chest radiograph findings have been divided into four categories: normal, typical, indeterminate and atypical [18]. Radiologists and/or pediatric pulmonologists reviewed all chest radiographs to ensure accuracy. The disease severity was classified as asymptomatic, mild (mild symptoms without pneumonia), moderate to severe (pneumonia with and without desaturation or no symptoms but abnormal chest radiograph), or critically ill (patients requiring mechanical ventilation or vasoactive medication; acute respiratory distress syndrome; or septic shock) [19].

### Statistical analysis

All data were entered into a Microsoft Excel database. The statistical analyses were performed using STATA software version 10 (Stata Corp., College Station, TX, USA). The categorical variables were summarized as counts and percentages (%). Continuous variables were assessed for normality using Shapiro-Wilk test and described as mean and standard deviation (SD) or median and interquartile range (IQR) depending on the normality of variables. We compared demographic data between pre-Omicron and Omicron cohort using chi-square or Fisher’s exact tests for categorical variables and independent t-test or Mann-Whitney U test for continuous variables. Values of *p* < 0.05 (two-tailed) were considered to indicate statistical significance between 2 groups. Multivariable linear regressions with backward stepwise elimination were used to demonstrate the mean difference of SARS-CoV-2 antibody levels between pre-Omicron and Omicron era with a 95% confidence interval (CI). Variables that have been shown to be associated with difference of SARS-CoV-2 antibody levels in bivariate analysis at a significance level of *p* < 0.2 were used as covariates.

### Ethics

This study was conducted with approval from the institutional review board of Khon Kaen University, Human Research Ethics Committee (#HE651444). The informed consent was waived as it was a retrospective observational study with no more than minimal risk.

## Results

### Studied population and characteristics

There were 119 hospitalized children due to COVID-19 included in our study, of whom 59.66% (n = 71) were male. The median age and interquartile range (IQR) of children was 49 months (17–87). All of them were confirmed COVID-19 by rtPCR. Twenty-four children (20.17%) had at least one co-morbidity. Asthma and allergic rhinitis were the most common accounted for 7 and 6 children, respectively. Eight asymptomatic patients were admitted with a high-risk condition (asthma, congenital heart disease, morbid obesity) who required close monitoring. A total of 83 cases (69.74%) had pulmonary infiltration from chest radiograph. They were diagnosed as COVID-19 pneumonia and classified as moderate to severe COVID-19. Only 2 of them had critically ill severity. Most of our patients received favipiravir as an antiviral therapy. All of the cases were unvaccinated against COVID. Fifty-eight children were infected during pre-Omicron, and 61 children were infected during Omicron era. The gender, comorbidities, chest radiograph classification were not statistically different. The median age of children during Omicron were significantly lower than pre-Omicron era (69 and 38 months, *p* = 0.009) (Table 1).

**Table 1:** Baseline characteristics of children infected with COVID-19 during pre-Omicron and Omicron era.

Characteristics	Total (n = 119)	Timing of infection		p-value
		Pre-Omicron (n = 58)	Omicron (n = 61)	
**Gender**				0.821
Male	71 (59.66)	34 (58.62)	37 (60.66)	
Female	48 (40.34)	24 (41.38)	24 (39.34)	
**Age (months)**				0.009
Median (IQR)	49 (17–87)	69 (31–123)	38 (12–71)	
**Comorbidities**				0.218
No	95 (79.83)	49 (84.48)	46 (75.41)	
Yes	24 (20.17)	9 (15.52)	15 (24.59)	
**PCR for SAR-CoV-2**				
ORF gene, Median (IQR)	22.02 (19.64–26.20)	22.5 (20.23–29.5)	21.63 (19.58–24.705)	0.141
N gene, Median (IQR)	19.68 (16.88–25.12)	19.92 (16.11–26.53)	19.49 (17.46–22.77)	0.836
E gene, Mean ± SD.	19.51 ± 5.39	18.20 ± 6.42	19.91 ± 5.37	0.651
RdRp gene, Mean ± SD.	21.69 ± 5.68	26.08 ± 3.31	20.44 ± 5.74	0.239
**Chest radiograph classification**				0.051
Negative study	34 (28.57)	12 (20.69)	22 (36.07)	
Intermediate	57 (47.90)	33 (56.90)	24 (39.34)	
Typical	28 (23.53)	13 (22.41)	15 (24.59)	
**Disease severity**				<0.001
Asymptomatic	4 (3.36)	4 (6.90)	0	
Mild	30 (25.21)	8 (13.79)	22 (36.07)	
Moderate to severe	83 (69.75)	46 (79.31)	37 (60.6)	
Critically ill	2 (1.68)	0	2 (3.28)	
**Treatment**				
Favipiravir	110 (92.44)	49 (84.48)	61 (100.00)	0.001
Steroid	2 (1.68)	0	2 (3.28)	0.496
Oxygen therapy	3 (2.54)	2 (3.45)	1 (1.67)	0.615

### COVID-19 infection in Omicron era is associated with lower platelet count but higher C-reactive protein level compared with pre-Omicron

The blood investigation results during hospitalization were presented in Table 2. The hemoglobin, and total white blood cell count were similar in both groups. The median platelet count in children infected during pre-Omicron era was higher than in children infected during the Omicron era (324,000 and 283,000, *p* = 0.008). The median level of C-reactive protein (CRP) in children infected during Omicron era was significantly higher than the median level of CRP during pre-Omicron era (*p* = 0.023).

**Table 2:** Serum levels of complete blood count, C-reactive protein, and COVID-19 antibody at diagnosis in children infected with COVID-19 during pre-Omicron and Omicron era.

Characteristics	Timing of infection		p value
	Pre-Omicron (n = 58)	Omicron (n = 61)	
**CBC**			
Hb (g/dL), Median (IQR)	12.3 (11.3–12.7)	12 (11.3–12.6)	0.317
Platelet (/uL), Median (IQR)	324000 (259000–428000)	283000 (245000–326000)	0.008
WBC (/uL), Median (IQR)	6445 (5180–9370)	7170 (5830–9300)	0.462
**CRP (mg/L), Median (IQR)**	0.65 (0.23–1.95)	1.33 (0.47–2.89)	0.023
**BUN (mg/dL), Mean ± SD.**	11.88 ± 3.21	11.32 ± 3.42	0.358
**Cr, Median (IQR)**	0.49 (0.39–0.60)	0.42 (0.32–0.49)	0.007

**Abbreviations:** BUN, Blood Urea Nitrogen; CBC, complete blood count; CRP, C-reactive protein; Cr, Creatinine; Hb, Hemoglobin; WBC, White blood cell count.

### COVID-19 infection in Omicron era associated with lower level of SARS-CoV-2 antibody compared with pre-Omicron

**Fig 1:**
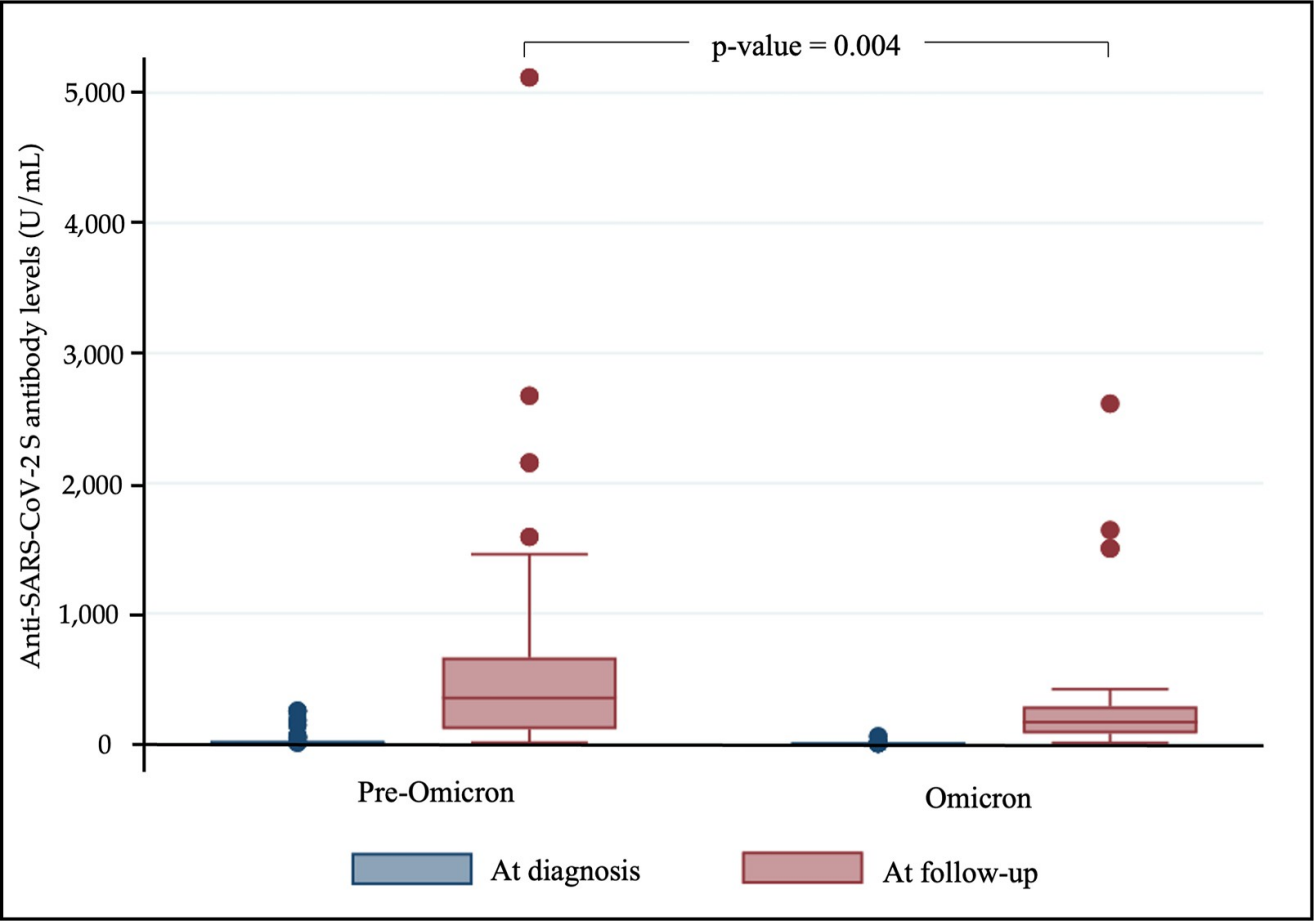
Anti-SARS-CoV-2 levels at diagnosis and follow-up in children infected with COVID-19 during pre-Omicron and Omicron era.

The median (IQR) of SARS-CoV-2 antibody levels at the time of diagnosis was 0.40 (0.40–3.00) U/mL during pre-Omicron and 0.40 (0.40–0.40) U/mL during Omicron. The overall median time (IQR) of follow-up was 79 days (72–95 days), which divided into the 77.5 days (72–86) and 88 days (74–104) after diagnosis, for children infected during pre-Omicron and Omicron era, respectively. At follow-up time, children infected during pre-Omicron and Omicron remained seropositive with the median of anti-SARS-CoV-2 antibody level of 206.1 (87.9–424.1) U/mL. The median level of Anti-SARS-CoV-2 antibody at follow-up of the children infected during pre-Omicron was 350.20 (IQR: 111.50–661.20) U/mL and that of children infected during Omicron was 165.20 (IQR 78.20–284.80) U/mL (*p* = 0.004) as shown in Fig 1. Children infected during pre-Omicron had mean of Anti-SARS-CoV-2 antibody levels at follow-up significantly higher than children infected during Omicron [mean difference (MD) = 292.57, 95% CI: 53.85–531.28, *p* = 0.017). No difference in mean of Anti-SARS-CoV-2 levels was found when the study participants were compared based on gender, age, co-morbidities, chest radiograph classification, and diagnosis (Table 3). The regression model adjusting for the covariates confirmed a significant difference (*p* = 0.017) in the Anti-SARS-CoV-2 antibody levels between children infected during pre-Omicron and Omicron.

**Table 3:** Factors associated with SARS-CoV-2 antibody level of children infected with COVID-19 at follow-up.

Factors	Total participants (n)	Mean (SD)	Mean difference	95%CI	p-value
**Era of COVID-19**					0.017
Omicron	61	278.38 (441.41)	Reference		
Pre-Omicron	58	570.94 (825.56)	292.57	53.85 to 531.28	
**Gender**					0.335
Female	48	348.54 (743.08)	Reference		
Male	71	469.94 (617.71)	121.39	-126.89 to 369.67	
**Age**					0.232
< 60 months	67	485.87 (754.90)	Reference		
≥ 60 months	52	337.35 (539.17)	-148.52	-393.56 to 96.51	
**Comorbidities**					0.204
No	95	381.57 (485.47)	Reference		
Yes	24	576.96 (1145.85)	195.39	-107.26 to 498.05	
**CXR classification**					0.340
Negative study	37	286.06 (481.52)	Reference		
Indeterminate	57	480.48 (790.96)	194.42	-85.83 to 474.67	
Typical	25	484.95 (602.92)	198.89	-144.78 to 542.56	
**Diagnosis**					0.479
Asymptomatic	4	330.60 (293.32)	Reference		
Mild	32	279.72 (511.21)	-50.88	-756.96 to 655.20	
Moderate to severe	81	486.90 (738.16)	156.30	-525.64 to 838.23	
Critically ill	2	191.85 (77.85)	-138.75	-1291.78 to 1014.28	

## Discussion

In this observational study, the level of SARS-CoV-2 antibodies in unvaccinated hospitalized children due to COVID-19 during pre-Omicron and Omicron era. Here, we found that children infected during Omicron era had lower SARS-CoV-2 antibody levels compared to pre-Omicron era. A previous study suggested that Omicron SARS-CoV-2 virus has lower virulence and lower activation of the host immune response [20,21]. Alternatively, the strains in pre-Omicron, especially the Delta variant showed more severe symptom and higher immune activation [22,23]. In our study, we found that the antibody levels at diagnosis during pre-Omicron was slightly greater than Omicron. The potential for confounding the antibody levels determined using the Elecsys assay exists due to the incongruity between the antigen utilized to quantify RBD antibodies and the infecting Omicron variants. As stated, the double-antigen principle of the Elycsys assay introduces a bias towards high-affinity antibody binding results when employing the receptor binding domain of the index strain. The pre-omicron era was characterized by the preponderance of the Beta and Delta variants, which each had a mere three and two RBD mutations, respectively. On the contrary, the preponderant Omicron BA.1, BA.2, and BA.5 variants exhibited a total of thirteen, sixteen, and seventeen RBD mutations during the studied period [24]. Consequently, antibodies generated against the pathogenic Omicron variant may potentially encounter numerous mismatches and fail to bind to the index spike RBD protein with significant affinity. So, the inquiry arises as to whether the antibody levels induced by Omicron are diminished, or whether the assay’s measurement of diminished binding is the result of discrepancies with the antigen utilized in the assay. At follow up, children infected during pre-Omicron had SARS-CoV-2 antibody levels higher than children infected during Omicron. This finding indicates that the increased immune activation was also present at the follow-up, highlighting that immune activation in Omicron is lower than pre-Omicron.

In our study, we also found that the presence of SARS-CoV-2 antibodies was not associated with the severity of illness. This result contradicts a prior study conducted on adults during the era of the delta variant, which demonstrated that elevated levels of SARS-CoV-2 neutralizing antibodies were associated with greater severity [25]. A study in adult showed that more severe COVID-19 infections resulted in a more robust humoral immune response, this finding may indicate that the mild illness in the population of children may not produce strong immune response [26]. It is also important to note that the presence of SARS-CoV-2 antibodies does not necessarily correlate with protection against future infections, several studies have shown that T-cell responses and memory B-cells may also play a role in immune protection against COVID-19 [27–32].

Several studies compared the SARS-CoV-2 neutralizing antibodies before and after vaccination [33,34]. Few studies investigated the unvaccinated children population. Comparing the initial diagnosis and post-infection levels of SARS-CoV-2 neutralizing antibodies in both pre-omicron and omicron era, we discovered that their presence is extremely low in our study. In contrast to the results in previous study of post vaccinated SARS-CoV-2 neutralizing antibodies in all kind of vaccines, they showed the higher level of SARS-CoV-2 antibodies [35]. A previous study has shown that COVID-19 neutralizing antibodies are generally lower in children following a natural infection with SARS-CoV-2 compared to vaccination with mRNA COVID-19 vaccines. This may be due to differences in the immune response between natural infection and vaccination [15]. This difference in the level of SARS-CoV-2 antibodies between natural infection and vaccination may be due to the composition and concentration of the antigen in the vaccine which is specifically designed to stimulate a strong and targeted immune response [36]. In contrast, natural infection with SARS-CoV-2 can result in a less targeted and weaker response, which may explain the lower levels of COVID-19 antibodies [13]. Despite the development of SARS-CoV-2 antibodies in unvaccinated children who have had COVID-19 infections, spontaneous infection in children may not provide adequate immunity to prevent subsequent infections, and vaccination remains the primary preventative approach [37]. This finding highlights the need for vaccination even, after natural infection as the immune response from natural infection has been shown not to be robust [38].

COVID-19 antibodies test can provide several benefits to children who have been exposed to or infected with SARS-CoV-2. Measuring COVID-19 antibodies in children can help determine whether they have been exposed to the virus, even if they were asymptomatic or had mild symptoms [39]. This is important for identifying infected individuals, tracking the spread of the virus, and making decisions about public health measures like quarantine and contact tracing [40]. COVID-19 antibodies test could be one key to identify MIS-C. This is a rare but severe condition that has been observed in some children who tested positive for COVID-19. One way to identify MIS-C is by testing for COVID-19 antibodies in unvaccinated children who have been affected by the virus [41]. Children who develop MIS-C typically have high levels of COVID-19 antibodies, which indicate that their immune system has been activated to fight the virus. Therefore, measuring COVID-19 antibodies could be a valuable tool in identifying children at risk for developing MIS-C [42]. Testing for COVID-19 antibodies in children with suspected MIS-C can improve the diagnostic accuracy and provide an optimal time for early medical intervention.

Our findings also demonstrate a statistical difference in CRP and platelet count in Omicron group vs pre-Omicron group. However, the levels of CRP and platelet were still within the normal range. Therefore, this difference appears not to be clinically significant. We also discovered a difference in the age group of Omicron variant that is lower than delta, which could suggest that the Omicron variants are more contagious [43].

Our study has some limitations that should be mentioned. For the recruited participants, the subjects were mild to moderate cases, no participant had severe illness hence it can be found less antibodies response in our population. Although our study is a retrospective study, standardized measurement protocols were employed in our study to ensure the collection of comprehensive data with minimal missing values. Furthermore, the strain type of virus of the participants are unknown. Age of the subjects between groups was significantly different. This factor might affect the immune status and the SARS-CoV-2 antibody level. Future study with multicenter approach to enroll more population with detailed viral strains is suggested.

## Conclusion

Our study showed that the host immune response in Omicron is different compared to pre-Omicron era by showing lower the SARS-CoV-2 antibody level. The Omicron seems to infect young children. Children do develop low level of SARS-CoV-2 antibodies in response to natural infection with SARS-CoV-2. These findings suggest that vaccine prioritization should be directed towards unvaccinated children. The immunization strategies and policies for children, particularly in areas where vaccine access is limited should be implemented worldwide.
